# Bacterial isolates and antibiotic susceptibility among women with abnormal vaginal discharge attending the gynecology clinic at a tertiary hospital in southwestern Uganda: a cross-sectional study

**DOI:** 10.1186/s12905-023-02746-w

**Published:** 2023-11-06

**Authors:** Onesmus Magezi Ahabwe, Taseera Kabanda, Lenard Abesiga, Julius Mugisha, Musa Kayondo, Joseph Ngonzi, Rodgers Tugume, Collins David Agaba, Onesmus Byamukama, Leevan Tibaijuka, Henry Mark Lugobe

**Affiliations:** 1 Department of Obstetrics and Gynecology, Faculty of Medicine, Mbarara University of Science and Technology, Mbarara, Uganda; 2 Department of Microbiology, Faculty of Medicine, Mbarara University of Science and Technology, Mbarara, Uganda

**Keywords:** Abnormal vaginal discharge, Antibiotic susceptibility, Bacterial isolates, Antimicrobial resistance, Mbarara

## Abstract

**Background:**

Abnormal vaginal discharge is a common complaint among women of reproductive age, affecting about one- third of all women. In resource-limited settings where access to laboratory services is limited, treatment is usually syndromic. This approach may result in ineffective treatment, with high recurrence rates and a potential of developing antibiotic resistance. This study aimed to determine the bacterial isolates and antibiotic susceptibility among women with an abnormal vaginal discharge attending the gynecology clinic at a tertiary hospital in Southwestern Uganda.

**Methods:**

We conducted a hospital based cross-sectional study among 361 women aged 15–49 years, presenting with abnormal vaginal discharge at the gynecology clinic of Mbarara Regional Referral Hospital from December 2020 to June 2021. Demographic characteristics were collected using a structured questionnaire. We collected cervical and vaginal sterile swabs and subjected them to wet preparation and gram stain. The specimens were cultured for bacterial isolates. Susceptibility testing was performed on samples with bacterial isolates using the Kirby-Bauer disc diffusion method, on the commonly prescribed antibiotics in this setting. We summarized and described the bacterial isolates and antibiotic susceptibility patterns as frequencies and percentages.

**Results:**

We enrolled 361 women with abnormal vaginal discharge. Bacteria were isolated in 29.6% (107/361) of the women, and the commonest isolates included; *Staphylococcus aureus* 48.6% (52/107), *Klebsiella pneumoniae* 29.9% (32/107) and *Enterococcus faecalis* 15% (16/107). Yeast cells were found in 17.7% (64/361) of the women with abnormal vaginal discharge. Cefuroxime (90.7%) and Ciprofloxacin (81.3%) had a high level of sensitivity while high levels of resistance were observed for Doxycycline (86.0%) and Azithromycin (67.0%).

**Conclusion:**

The common bacterial isolates were *Staphylococcus aureus*, *Klebsiella pneumoniae* and *Enterococcus faecalis*. The isolated bacteria were most sensitive to Cefuroxime and Ciprofloxacin but resistant to Doxycycline and Azithromycin. There is need for routine culture and susceptibility testing of women with abnormal vaginal discharge so as to guide treatment, minimize inappropriate antibiotic use and consequently reduce antibiotic resistance.

## Background

Abnormal vaginal discharge is a frequent complaint among patients attending obstetrics and gynecology clinics, occurring in approximately one-third of all women and half of pregnant women [1, 2]. The symptom is usually the first evidence of genital tract infections, and these infections may result in gynecological complications. These include pelvic inflammatory disease (PID) with subsequent tubal factor infertility, ectopic pregnancy, adverse pregnancy outcomes (such as spontaneous abortions, preterm delivery, low birth weight), cervical dysplasia, and increased risk of postoperative infection [3], amplifying HIV and HSV-2 acquisition and transmission [3, 4].

Treatment in this setting is usually syndromic using cefixime and doxycycline as the recommended first-line antibiotics [5], without routine culture and sensitivity tests, yet antibiotic susceptibility patterns change over time. There is a paucity of literature in our setting about the susceptibility of bacteria isolated in women with abnormal vaginal discharge to these antibiotics. The use of multiple drug combinations in syndromic treatment, without routine culture and sensitivity testing, has the potential to promote the already growing concern of antibiotic resistance with low cure and high recurrence rates.

This study therefore aimed to determine the bacterial isolates and their antibiotic susceptibility among women with abnormal vaginal discharge attending the gynecology clinic of a tertiary hospital in southwestern Uganda.

## Methods

### Study design, setting, and study population

We conducted a cross-sectional study at the gynecology clinic and microbiology laboratory of Mbarara Regional Referral Hospital (MRRH) from December 2020 to June 2021 among reproductive-age women (15–49 years) with abnormal vaginal discharge. MRRH is a tertiary hospital located in Mbarara City, southwestern Uganda, approximately 250 km from Kampala, the capital city. MRRH is the main referral hospital for all of southwestern Uganda, serving over 10 districts and receiving patients from the neighboring countries of Tanzania, Rwanda, the Democratic Republic of Congo (DRC) and Burundi.

### Sample size and sampling technique

We used the Kish Leslie formula of 1965 for calculating the sample size for cross-sectional studies and obtained 361 as the minimum number of respondents, whom we enrolled using consecutive sampling. We calculated the sample size using a population proportion of 70% women with abnormal virginal discharge based on a study from Nigeria [6] to have isolates.

### Study procedure

All women aged 15–49 years attending the gynecology clinic of MRRH (from December 2020 to June 2021) with a complaint of abnormal vaginal discharge with or without other symptoms were approached to participate in the study. We included all non-pregnant women, while women who also had vaginal bleeding and those with confirmed genital malignancy were excluded. The research team screened the women for eligibility and obtained written consent from the eligible participants. Gynecology examination was performed for all participants enrolled in the study. The participants underwent a speculum examination during which two endocervical and two high vaginal swab samples were collected. A standardized interviewer-guided structured questionnaire was used to obtain data on sociodemographic, sexual, obstetric, gynecological and medical characteristics of the participant, including HIV serostatus.

The women received their empirical treatment as guided by the syndromic management of abnormal vaginal discharge as per the clinical care guidelines [5] and were asked to return after three days for their culture and susceptibility results. Participants with bacterial isolates and susceptibility tests were linked to the clinical care team for further management.

### Specimen collection

The patient was in the lithotomy position during sample collection. The principal investigator or attending physician swabbed the vulva with cotton swabs that were premoistened with normal saline, separated the patient’s labia minora and majora with one hand, and gently introduced a sterile Cusco’s speculum into the patient’s vagina to visualize the cervix.

With a Cusco’s speculum in situ and under direct visualization of the cervix, the endocervix was swabbed using a sterile cotton swab by inserting the cotton swab 20–30 mm into the cervical canal and rotating the swab gently through 360 degrees clockwise against the endocervical wall. Two swabs were collected with minimal interference: one for culture and susceptibility and the other for Gram staining.

To collect the vaginal specimen, sterile cotton swabs were inserted and rotated 360 degrees onto the posterior vaginal fornix under direct vision. Two swabs were collected. One was used for wet preparation and gram staining, and the other was used for culture and sensitivity.

Each of the swabs collected was inserted back into the tube from which it had been taken, labeled with the participant’s study number, initials, date, and time of collection, and then transported to the laboratory in a cold box.

A bimanual examination was then performed by the principal investigator/attending resident/specialist in all patients to look for adnexal tenderness or any other pathology as part of the routine gynecological examination of the patients.

### Sample transportation and handling

The swabs were transported by a member of the research team within 30 min of collection to the medical microbiology laboratory of Mbarara University. If the sample transport was delayed, the swabs were placed in Carry Blaire transport medium in a cool box.

In the laboratory, the samples were subjected to:

#### Wet preparation

This was done to observe *Trichomonas vaginalis* and budding yeast cells. The samples from the endocervical canal and vagina were separately emulsified in sterile normal saline, and a drop of these mixtures was placed on separate slides using a sterile pipette and viewed under a × 40 objective lens to look for motile Trichomonas vaginalis and budding yeast cells.

#### Gram stain

Specimen slides from each participant: swabs from both the endocervix and posterior vaginal fornix were prepared, fixed, Gram stained, and examined under oil immersion at × 100 for the presence of granulocytes, clue cells (vaginal epithelial cells with a granular surface and blurred margins because of attached bacteria indicative of bacterial vaginosis) and gram-positive budding yeast cells. The procedure involved labeling the frosted end of the slide with the patient number using a lead pencil. A cotton swab with the specimen was then rolled on the slide and spread evenly covering a 15–20 mm diameter on the slide. The slide was then air-dried and fixed with 2 drops of 50% acetone alcohol and left for two minutes for alcohol to dry. The fixed smear was then covered with crystal violet stain for 30–60 s. The stain was then washed off with clean water, all the water tipped off, and then the smear was covered with Lugol’s iodine for 30–60 s, after which the iodine was washed off. The smear was then rapidly decolorized using acetone-alcohol, washed with clean water, and covered with neutral red stain for 2 min, after which the stain was washed off and the slide air-dried. Then, the smear material was initially examined under a microscope under a × 40 objective lens to check for staining and distribution, after which it was examined under an oil immersion objective lens to report the bacteria and cells. Gram-positive bacteria and yeast cells appear dark purple, while gram-negative bacteria and nuclei of pus cells appear red.

#### Culture

Endocervical and vaginal specimens for culture were inoculated onto 5% sheep blood agar, MacConkey agar, mannitol salt agar, and modified Thayer Martin agar to isolate aerobic bacteria. The inoculated media was incubated at 37 °C aerobically for 24–72 h. Modified Thayer Martin agar plates were incubated in a humidified atmosphere with 5% filtered carbon dioxide. Identification of the cultured isolate was performed by conventional phenotypic and biochemical methods, which included catalase, coagulase, and DNase for Staphylococcus aureus (which produces positive catalase, coagulase, and DNase tests) and urease, citrate utilization, oxidase, and triple sugar iron for identification and differentiation of gram-negative bacilli.

#### Antimicrobial susceptibility testing

This was done on a pure culture showing significant growth following standard criteria as described by the Kirby-Bauer disc diffusion method [7]. The medium for fastidious organisms was chocolate agar incubated in carbon dioxide. For nonfastidious organisms, we used Muller Hinton Agar (MHA) incubated aerobically at 37°C. The inoculum density required for susceptibility testing was 0.5%, McFarland. The choice of antibiotic discs was based on the type of organism(s) cultured. The following antimicrobial agents were employed: tetracycline (50 μg), ceftriaxone (30 μg), ciprofloxacin (5 μg), amoxicillin (10 μg), and erythromycin (15 μg) for gram-positive organisms and gentamycin (10 μg), amoxicillin/clavulanate, cefixime, cefuroxime, azithromycin, and doxycycline for susceptibility testing for gram-negative organisms. Susceptibility testing for erythromycin was only performed against gram-positive bacteria, as the antibiotic is not active against gram-negative bacteria. Also susceptibility testing for *S. agalactiae* against Ciprofloxacin and *Klebsiella pneumoniae* against Amoxicillin were not done as these antibiotics are not active against these isolates.

#### Procedure

Using a sterile wire loop, colonies of pure bacterial growth were scraped from the culture plate bearing the cultured organism and suspended in 2 ml of sterile peptone water to make a suspension of 0.5 McFarland standard. A sterile cotton swab was then immersed in the suspension of peptone water containing the test organism, and excess peptone water was squeezed out by pressing the cotton swab against the wall of the tube above the level of the suspension. The cotton swab containing the test organism was then used to uniformly inoculate the Mueller Hinton agar plate that would have been dried for 15 min at 37 °C in an incubator. The same was also done for the suspension of the standard organisms each time a sensitivity test was performed. The suspension of the standard organisms that was being used for internal quality control was *S. aureus* ATCC25923 for gram-positive bacteria and *E. coli* ATCC25922 for gram-negative bacteria.

Five antibiotic discs were then placed on the surface of the agar plate using sterile forceps at a distance of at least 2.5 cm from each other, and the culture plates were incubated at 37°C aerobically for 24–48 h. The diameters of zones of growth inhibition around each antibiotic disc were measured in millimeters using a ruler and compared against the diameter of zones of growth inhibition of the standard organism following the Clinical Laboratory Standards Institute – USA- 2020.

### Data analysis

Data were entered into the REDCap ™ database and exported to STATA version 15 for cleaning and statistical analysis. Descriptive statistics of participant characteristics were summarized using mean and standard deviation for continuous variables and frequencies for the categorical variables and then presented in tables. Isolated and identified microorganisms are presented on a bar graph with each quantified as a percentage of total isolated organisms. The susceptibility patterns of the bacterial isolates to the tested antibiotics were expressed as frequencies and percentages.

## Results

A total of 373 women of reproductive age presented at the gynecology clinic of Mbarara Regional Referral Hospital with abnormal vaginal discharge during the study period. Of these, 12 were excluded because they had concurrent vaginal bleeding. Culture was performed on swabs collected from 361 of these women, and bacteria were isolated in 107 of the participants. The mean age of the participants was 36.42 (± 7.99) years, as shown in Table 1 below. There was no significant difference in the sociodemographic characteristics between women with and without bacterial isolates.

**Table 1:** Participants’ sociodemographic characteristics, *N* = 361

Variable	n (%)	Bacteria Isolated		*P* value
		**Yes, *N* = 107 n (%)**	**No, *N* = 254 n (%)**	
**Age Mean ± SD**	36.42 (± 7.99) years	37 ± 7.67 yrs	36.01 ± 8.11yrs	0.933
**Residence**				0.948
Urban	161 (44.6)	48 (44.86)	113	
Rural	200 (55.4)	59 (55.14)	141	
**Education level**				0.814
Primary and below	216 (59.8)	66 (61.68)	160 (62.99)	
Secondary and above	135 (40.2)	41 (38.32)	94 (37.01)	
**Occupation**				0.245
Unemployed	223 (61.8)	71 (66.36)	152 (59.84)	
Employed	138 (38.2)	36 (33.64)	102(40.16)	
**HIV status**				0.365
Positive	106 (29.4)	35 (32.71)	71 (27.95)	
Negative	255 (70.6	72 (67.29)	183 (72.05)	
**Marital status**				0.862
Living with partner	252 (69.8)	74 (69.16)	178 (70.08)	
Not living with partner	109 (30.2)	33 (30.84)	76 (29.92)	
**Sexually active in last 6 months**				0.873
No	49 (13.6)	15 (14.02)	34 (13.39)	
Yes	312 (86.4)	92 (85.98)	220 (86.61)	

*HIV* Human Immunodeficiency Virus, *yrs* years

Prior infertility treatment and duration of abnormal vaginal discharge differed among participants with and without bacterial isolates. Bacterial isolates were more abundant in participants who had not received prior treatment for infertility than in those who had received the treatment. The participants with a shorter duration (≤ 7 days) of abnormal vaginal discharge were more likely to have bacteria isolated than those with a longer duration of abnormal vaginal discharge, as shown in Table 2.

**Table 2:** Gynecological and medical characteristics of the study participants, *N* = 361

Variable	n (%)	Bacteria Isolated		*P* value
		**Yes, *N* = 107 n (%)**	**No, N = 254 *n* (%)**	
**Prior infertility treatment**				**0.199**
Yes	14 (3.9)	2 (1.87)	12 (4.72)	
No	347(96.1)	105 (98.13)	242 (95.28)	
**Barrier FP method (condoms) *n* = 112**				**0.235**
Yes	8 (2.2)	4 (11.43)	4 (5.19)	
No	104 (28.8)	31(88.57)	73 (94.81)	
**Miscarriage in prior 6 weeks**				**0.598**
Yes	13 (3.6)	3 (2.80)	10 (3.94)	
No	348 (96.4)	104 (97.20)	244 (96.06)	
**Prior abnormal vaginal discharge**				**0.479**
Yes	259 (71.7)	74 (69.16)	185 (72.83)	
No	102 (28.3)	33 (30.84)	69 (27.17)	
**AVD times in last 6 months**				**0.324**
None	102 (28.3)	33 (30.84)	69 (27.17)	
Once	137 (38.0)	44 (41.12)	93 (36.61)	
> = two times	122 (33.8)	30 (28.04)	92 (36.22)	
**Duration of current abnormal discharge**				**0.02**
≤ 7 days	161 (44.6)	45 (42.06)	116 (45.67)	
8–14 days	104 (28.8)	41 (38.32)	63 (24.8)	
More than 14 days	96 (26.6)	21 (19.63)	75 (29.53)	
**Prior antibiotic use**				0.705
Yes	93 (25.8)	29 (27.1)	64 (25.2)	
No	268 (74.2)	78 (72.9)	190 (74.8)	
**Long term drug**^**a**^** use**				0.499
Yes	109 (30.2)	35 (32.71)	74 (29.13)	
No	252 (69.8)	72 (67.29)	180 (70.87)	

*FP* Family Planning, *AVD* Abnormal Vaginal Discharge

^a^ Refers to use of antihypertensive or hypoglycemic drugs

**Fig. 1:**
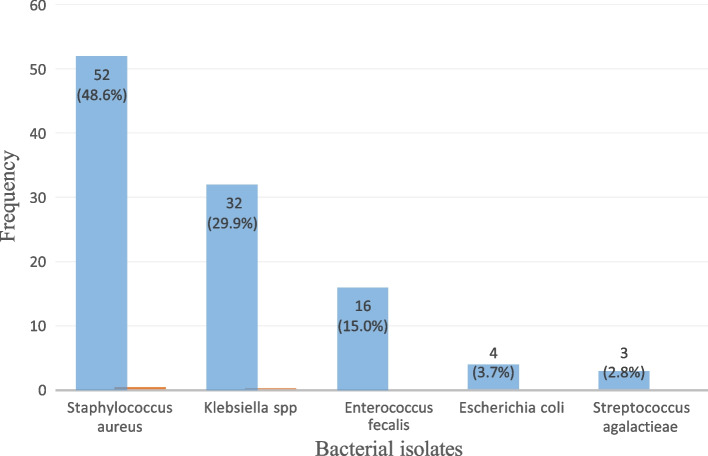
Bar graph showing bacterial isolates from women with abnormal vaginal discharge (*n* = 107)

Bacteria were isolated from endocervical and high vaginal swab samples from 107 participants. *Staphylococcus aureus* was the most common isolate, followed by *Klebsiella pneumoniae* and *Enterococcus faecalis,* as shown in Fig. 1*. Staphylococcus aureus, Enterococcus faecalis* and *Streptococcus agalactiae* are gram-positive bacteria, while *Klebsiella pneumoniae* and *Escherichia coli* are gram-negative bacteria.

The total sensitivity of all bacterial isolates was highest with cefuroxime at 90.7% and ciprofloxacin at 81.3%. The highest resistance was observed with doxycycline at 86%, as shown in Table 3.

**Table 3:** Antibiotic susceptibility of the bacterial isolates

Antibiotic		Bacterial isolates n (%)					Total *N* = 107
		*S.aureus n* = 52(%)	*S.agalactieae n* = 3 (%)	*Kleb spp n* = 32 (%)	*E.coli n* = 4 (%)	*E. fecalis n* = 16 (%)	
Amoxicillin	S	22(42.3)	2(66.7)	Not tested	2(50)	13(81.3)	39 (36.5)
	R	30(57.7)	1(33.3)	Not tested	2(50)	3(18.7)	68 (63.5)
Amoxicillin/Clavulanate	S	41(78.8)	3(100)	22(68.8)	4(100)	13(81.3)	83(77.6)
	R	11(21.2)	0(00)	10(31.2)	0(00)	3(18.7)	24(22.7)
Ciprofloxacin	S	42(80.8)	Not tested	26(81.3)	3(75)	16(100)	87 (81.3)
	R	10(19.2)	Not tested	6(18.7)	1(25)	0(00)	20 (18.7)
Ceftriaxone	S	46(88.5)	3(100)	3(9.4)	4(100)	12(75)	68 (63.6)
	R	6(11.5)	0(00)	29(90.6)	0(00)	4(25)	39 (26.4)
Cefixime	S	41(78.8)	3(100)	21(65.6)	3(75)	14(87.5)	82 (76.6)
	R	11(21.2)	0(00)	11(34.4)	1(25)	2(12.5)	25 (23.4)
Cefuroxime	S	48(92.3)	3(100)	27(84.4)	4(100)	15(93.8)	97 (90.7)
	R	4(7.7)	0(00)	5(15.6)	0(00)	1(6.2)	10 (9.3)
Erythromycin	S	11(21.2)	0(00)	Not tested	Not tested	3(18.7)	14 (19.7)
	R	41(78.8)	3(100)	Not Tested	Not tested	13(81.3)	57 (80.3)
Gentamycin	S	38(73.1)	3(100)	27(84.4)	3(75)	13(81.3)	84 (78.5)
	R	14(26.9)	0(00)	5(15.6)	1(25)	3(18.7)	23 (21.5)
Tetracycline	S	3(5.8)	1(33.3)	4(12.5)	0(00)	1(6.2)	9 (8.4)
	R	49(94.2)	2(66.7)	28(87.5)	4(100)	15(93.8)	98 (91.6)
Doxycycline	S	7(13.5)	1(33.3)	6(18.7)	0(00)	1(6.2)	15 (14)
	R	45(86.5)	2(66.7)	26(81.3)	4(100)	15(93.8)	92 (86)
Azithromycin	S	22(42.3)	2(66.7)	8(25)	2(50)	12(75)	46 (43)
	R	30(57.7)	1(33.3)	24(75)	2(50)	4(25)	61 (67)

*S* Sensitive, *R* Resistant, *Kleb spp* Klebsiella pneumoniae, *S. aureus* Staphylococcus aureus, *S. agalactiae* Streptococcus agalactiae, *E. coli* Escherichia coli, *E. faecalis* Enterococcus faecalis

## Discussion

Bacteria were isolated in 29.6% of the samples from women with abnormal vaginal discharge. The most common bacterial isolates were *Staphylococcus aureus (48.6%)*, *Klebsiella pneumoniae (29.9%)*, and *Enterococcus faecalis (15.0%)*. The isolates were most resistant to doxycycline (86.0%), azithromycin (67.0%), and cefixime (23.4%). The overall sensitivity across all bacterial isolates was highest to cefuroxime (90.7%) and ciprofloxacin (81.3%).

The proportion of samples in which bacteria were isolated in our study is similar to that found in studies from Nigeria and Uganda [6, 8], where bacteria were isolated in 28.0% and 27.4% of the samples, respectively. This could be because these studies were conducted in tertiary care facilities with similar settings. This proportion was, however, lower than that in other studies: 33.0% [9] in Nigeria, 38.0% [10] in Cameroon, 41.5% and 39.5% [11, 12] in Ethiopia, and 43.1% [13] in Kenya, which could be due to differences in participant characteristics.

The most common bacterial isolates were *Staphylococcus aureus*, *Klebsiella pneumoniae*, and *Enterococcus faecalis*. These bacterial isolates are similar to those isolated in other studies in Burkina Faso and Ethiopia [12, 14, 15], which are developing countries with similar settings. *Staphylococcus aureus* is a resident flora on the skin, including the perineum, and sexual activity increases the chances of transmission from the skin to the upper vagina and cervix [16, 17]. *Klebsiella pneumoniae and Enterococcus fecalis* are organisms that usually inhabit the gastrointestinal tract and are more commonly found in stool samples. The closer proximity of the female genital tract and the anal opening makes it easy for these organisms to spread to the genitourinary tract with sexual intercourse, facilitating this transmission [18].

The isolates were most resistant to doxycycline, azithromycin, and cefixime. This could be because these antibiotics are commonly used in treating women with abnormal vaginal discharge as per the Ministry of Health Uganda guidelines [5], and yet there is unregulated access to these drugs over the counter [19]. This promotes the development of antibiotic resistance by allowing genetic alterations that result in gene expression changes and mutations [20].

The overall sensitivity across all bacterial isolates was highest to cefuroxime and ciprofloxacin. Other antibiotic susceptibility studies conducted at Mbarara Regional Referral Hospital have shown similar sensitivity to ciprofloxacin [21, 22]. Similarly, high sensitivity to ciprofloxacin has also been reported at a tertiary care facility in Ethiopia [12]. The high sensitivity to ciprofloxacin and cefuroxime in our study could be because under the Ugandan clinical guidelines, ciprofloxacin and cefuroxime are not routinely used in the treatment of abnormal vaginal discharge [5]. Further research studies are needed to understand the susceptibility pattern to ciprofloxacin that has been observed in this setting and whether this drug could be an important treatment option.

Our study had some limitations. First, we might have underreported the bacterial isolates due to our inability to culture some bacteria such as Chlamydia. We were also not able to culture anaerobic organisms, which could have underestimated the samples with isolated organisms. Second, we acknowledge that fungal infections and parasitic infections whose isolation and investigation were beyond the scope of this study may have possibly contributed to the high proportion of clinically symptomatic participants from whom no bacterial pathogens were isolated.

## Conclusions

Bacteria were isolated in 3 of every 10 women with abnormal vaginal discharge. The majority of the bacterial isolates were resistant to antibiotics commonly used in the treatment of women with abnormal vaginal discharge at Mbarara Regional Referral Hospital. There is a need for routine culture and susceptibility testing of women with abnormal vaginal discharge to guide treatment, minimize inappropriate antibiotic use and consequently reduce antibiotic resistance.

## Abbreviations

ATCC: American Type Culture Collection
DNA: Deoxyribonucleic Acid
MRRH: Mbarara Regional Referral Hospital
HIV: Human Immunodeficiency Virus
HSV: Herpes Simplex Virus
MUST: Mbarara University of Science and Technology
REDCap: Research Electronic Data Capture
UNCST: Uganda National Council for Science and Technology

## Declarations

### Availability of data and materials

The datasets used and/or analyzed during the current study are available from the corresponding author upon reasonable request.
